# Ageing-related changes in the levels of β-catenin, CacyBP/SIP, galectin-3 and immunoproteasome subunit LMP7 in the heart of men

**DOI:** 10.1371/journal.pone.0229462

**Published:** 2020-03-02

**Authors:** Irena Kasacka, Żaneta Piotrowska, Michał Niezgoda, Alicja Lewandowska, Wojciech Łebkowski

**Affiliations:** 1 Department of Histology and Cytophysiology, Medical University of Białystok, Białystok, Poland; 2 Department of Neurosurgery, Medical University of Bialystok, Białystok, Poland; Universidade de Sao Paulo, BRAZIL

## Abstract

Aging is a major risk factor for morbidity and mortality from cardiovascular causes in men. To better understand the cellular processes related to age-related cardiac complications, we undertook research aimed at comparative evaluation of genes expression and distribution of β-catenin, CacyBP/SIP, galectin-3 and LMP7 in the heart of healthy men in different age groups. The study was conducted on the hearts of 12 men (organ donors) without a history of cardiovascular disease, who were divided into two age groups: men under and men over 45 years of age. On paraffin sections, immunohistochemical reactions were performed to detect β-catenin, CacyBP/SIP, galectin-3 and immunoproteasome subunit LMP7. The expression of genes coding β-catenin, CacyBP/SIP, galectin-3 and LMP7 was also evaluated by real-time PCR method. In the heart of men over 45 years old, both gene expression and immunoreactivity of β-catenin, CacyBP/SIP, galectin-3 and LMP7 were stronger compared to younger individuals. The results of the presented studies suggest that β-catenin, CacyBP/SIP, galectin-3 and immunoproteasomes might be involved in the internal regulation of heart homeostasis during ageing.

## Introduction

One aspect of aging of the entire human body is the aging of the heart and circulatory system. As the aging process progresses, the structure and function of the heart changes [1].

Despite continuous efforts by clinicians and scientists to increase the effectiveness of prophylaxis and treatment of cardiovascular complications in older people, cardiovascular diseases remain the main cause of mortality, accounting for over one third of all deaths [2]. A more detailed diagnosis of the intracellular mechanisms underlying the functional changes of cardiomyocytes is essential for a better understanding of the complex process of aging of the heart.

The β-catenin is a double-function protein in the cell. Cytoplasmic β-catenin determines cell function by modulating gene transcription. In contrast, membrane bound β-catenin coordinates cellular connections and is responsible for maintaining tissue architecture [3]. This protein is of key importance for cardiovascular system homeostasis. Several *in vivo* and *in vitro* studies have shown that β-catenin affects the differentiation and survival of myocardial cells [3–5]. β-catenin plays an important role in mechanical coherence and electrical coordination of cardiomyocytes, since it participates in creating adhesions and gap junctions in the intercalated discs as well as regulates the transmission of electrical signals through calcium and sodium channels [6–9]. β-catenin is also involved in hypertrophic, fibrotic and hemodynamic changes in the heart caused by myocardial infarction, ischemia-reperfusion injury, pressure/volume overload, angiotensin II-induced hypertension [3, 10–13].

As a transcription factor, β-catenin is tightly regulated by proteasomal degradation. In the classical pathway, β-catenin is phosphorylated by a dual-kinase mechanism involving casein kinase I (CKI) and glycogen synthase kinase 3β (GSK-3β) in the so-called β-catenin destruction complex. Subsequently phosphorylated β-catenin is ubiquitinated and undergoes proteasomal degradation [3, 14, 15]. Recently, an alternative pathway for the degradation of β-catenin with the participation of CacyBP/SIP protein (Calcyclin-binding protein/Siah-1-interacting protein) has been discovered [16]. CacyBP/SIP is a multi-domain protein that interacts with a wide range of intracellular molecules, including the components of ubiquitin ligases Siah-1 (seven in absentia homologue-1) and Skp1 (S-phase kinase associated protein 1). CacyBP/SIP combines with Siah-1 and Skp1, stabilizes the ubiquitin ligase complex and promotes the degradation of non-phosphorylated β-catenin [16].

To date, only few reports on the importance of CacyBP/SIP in the functioning of the cardiovascular system have been published and hence the current body of knowledge in this field is limited. Research on neonatal rats and cultured cardiac myoblasts (H9C2 cells) has demonstrated that CacyBP/SIP plays an important role in the differentiation of cardiomyocytes and heart development [17]. The same investigation revealed the protective effect of CacyBP/SIP on cardiomyocytes under stress of hypoxia-reoxygenation [17]. Our previous study showed an increase in CacyBP/SIP content in the heart of hypertensive rats of different etiology, which may suggest the involvement of CacyBP/SIP in hypertensive cardiac complications [18].

Recent literature reports indicated that β-catenin signalling is also regulated by galectin-3. Galectin-3 is a member of the galectin family, which comprises the peptides having at least one carbohydrate recognition domain and specific affinity for β-galactosides [19]. It has been found that galectin-3 has a structural similarity to β-catenin and is similarly phosphorylated by GSK-3β or CKI. Galectin-3 can lead to the accumulation of β-catenin in cells by simultaneously increasing the expression of β-catenin and inhibiting its proteasomal degradation. Galectin-3 also mediates the translocation of β-catenin to the nucleus and induces transcriptional activity [20, 21].

Galectin-3 is now considered one of the prognostic markers of heart failure and death from cardiovascular causes [22–25]. Increased levels of circulating galectin-3 have been found in the blood of patients with heart failure and individuals with cardiac hypertrophy, caused by aortic stenosis [22–25]. Elevated galectin-3 level has also been observed in the myocardium in experimental studies of heart disease such as myocardial infarction, Ren-2 rats prone to heart-failure, interferon-γ-induced cardiomyopathy, angiotensin II-induced hypertension, streptozotocin-induced diabetic cardiomyopathy and heart failure caused by pulmonary artery belts [22–25]. Numerous reports have indicated the participation of galectin-3 in myocardial fibrosis, myocarditis, hypertrophy of the heart muscle and ventricular dysfunction [22–25]. In addition, galectin-3 has a negative impact on the antioxidative capacity of cardiomyocytes and promotes the generation of reactive oxygen species, leading to increased apoptosis of myocardial cells [22, 26, 27].

Cellular senescence is accompanied by the impairment of numerous internal processes including disturbance in the balance between the synthesis and degradation of proteins in cells [28, 29]. One of the major mechanisms of selective protein degradation in cells are proteasomes which participate in the turnover of approximately 80–90% of proteins.

With age, the activity of proteasomes decreased, which leads to the accumulation of damaged or non-functional proteins in cells [28, 29]. The imbalance between levels of antioxidants and prooxidants inducing oxidative stress is one of the main causes of cellular aging [30]. The oxidative stress causes the oxidation of lipids and proteins as well as disturbances in DNA structure [30]. It has been suggested that following the oxidative modification during aging, the proteolytic capacity of the proteasomes decreases [28, 29]. Moreover, the increasing oxidative stress in aging cells might induce a change in constitutive proteasomes in immunoproteasomes. In immunoproteasomes, the standard β1, β2, and β5 subunits are replaced by β1i (LMP2), β2i (LMP10) and β5i (LMP7) the so-called immune subunits, respectively. Immunoproteasomes are less effective than constitutive ones in removing damaged proteins since they are predominantly involved in the generation of antigen peptides present in immune cells [31, 32].

To date, only the effect of aging on the content of β-catenin and proteasomal activity in the heart of laboratory animals has been investigated [29, 33–35] and only a few clinical trials demonstrating alterations in galectin-3 concentration in the blood of older individuals have been conducted [24, 25]. Due to ethical difficulties with obtaining material from healthy people, there are no reports describing changes in the levels of β-catenin, CacyBP/SIP, galectin-3 and proteasome activity in the aging human myocardium.

Considering the involvement of β-catenin, CacyBP/SIP and galectin-3 in the regulation of cardiac function, as well as the strict functional dependence of these proteins on proteasomal activity, the current study was performed to evaluate immunohistochemically and to compare gene expression of β-catenin, CacyBP / SIP, galectin-3 and immunoproteasome subunit LMP7 in the heart of healthy men at different ages.

## Material and methods

### Sample collection

Twelve adult men (organ donors) without history of cardiovascular disease were used in this study. The men were in age range from 21–65 years, mean body weight 84.5 ± 1.26 kg and mean BMI (body mass index) 26.3 ± 0.28 kg/m^2^. The men were divided into two groups: subjects older than 45 years (six men) and subjects under 45 years old (six men).

Each man presented with clinical symptoms of brain death was considered to be an organ donor. Irreversible brain damage was confirmed by special clinical examination and angiography (no blood flow within the brain arteries).

After diagnosis and confirmation of brain death and after collection of organs for transplantation (kidneys, liver), a heart sample was taken from each body and immediately fixed in 10% buffered formalin solution and routinely embedded in paraffin. Sections (4 μm) were stained with haematoxylin-eosin for general histological examination and processed by immunohistochemistry for detection of the β-catenin, CacyBP/SIP, galectin-3 and immunoproteasome subunit LMP7.

### Ethical issues

The study protocol was approved by the Ethics Committee, Medical University of Białystok (R-I-002/345/2007), and a written informed consent had previously been obtained from each man or from his family member(s).

### Immunohistochemistry

Paraffin blocks were cut into 4-μm sections (3 sections from each subject for each antibody) and attached to positively charged glass slides. Immunohistochemistry was performed, using an REAL^™^ EnVision^™^ Detection System, Peroxidase/DAB, Rabbit/Mouse detection kit (K5007; DakoCytomation) [36]. Immunostaining was performed according to the following protocol: paraffin-embedded sections were deparaffinized and hydrated in pure alcohols. For antigen retrieval, the sections were subjected to pre-treatment in a pressure chamber heated for 1 minute at 21 psi at 125°C (one pound force per square inch [1 psi] equates to 6.895 kPa, the conversion factor has been provided by the United Kingdom National Physical Laboratory). During antigen retrieval sections were incubated with Target Retrieval Solution Citrate pH = 6.0 S 2369 (DakoCytomation). After cooling down to room temperature, the sections were incubated with Peroxidase Blocking Reagent S 2001 (DakoCytomation) for 10 min to block endogenous peroxidase activity. Subsequently, the sections were incubated overnight with the primary antibodies against β-catenin (No ab32572 purchased from Abcam), CacyBP/SIP (obtained from the Nencki Institute of Experimental Biology, produced in-house as described in report by Jastrzębska et al. [37]), galectin-3 (No A3A12, purchased from Thermo Fisher Scientific) and LMP7 (No PW8845, purchased from Biomol) at 4°C in a humidified chamber.

**Fig 1:**
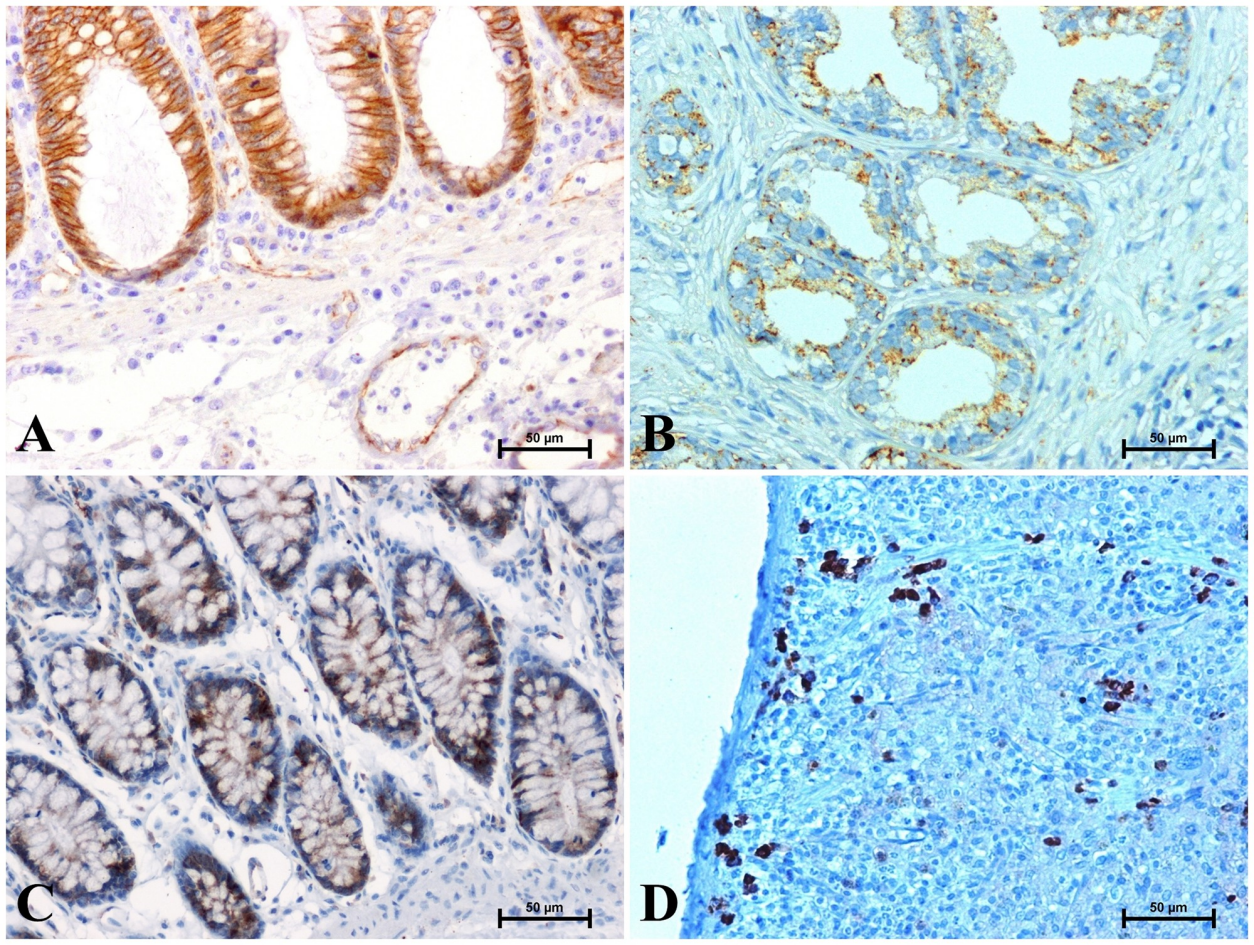
Positive test for β-catenin antibody (A) CacyBP/SIP antibody (B), galectin-3 antibody (C) and LMP7 antibody (D).

The antisera were previously diluted in Antibody Diluent (S 0809, DakoCytomation), in proportion 1:4000 for β-catenin, 1:50 for CacyBP/SIP, 1:1000 for galectin-3 and 1:5000 for LMP7. The procedure was followed by incubation with secondary antibody (conjugated to horseradish peroxidase-labelled polymer). Appropriate washes with S 3006 Wash Buffer (DakoCytomation) were performed between each step. The bound antibodies were visualized by 1 minute incubation with liquid 3,3^’^-diaminobenzidine (DAB) substrate chromogen. The sections were finally counterstained in QS haematoxylin (H-3404, Vector Laboratories, Burlingame, CA, USA), mounted and evaluated under light microscope. Specificity test, performed for the β-catenin, CacyBP/SIP, galectin-3 and LMP7 antibody included a negative and positive control. In negative control antibodies were replaced by normal rabbit serum (Vector Laboratories, Burlingame, CA, USA) at respective dilution. For negative control, no immunostaining was observed in heart tissues under the omission of the primary antibodies. Human tissues were a positive control: large intestine for β-catenin and galectin-3, prostate for CacyBP/SIP, and spleen for LMP7 (Fig 1).

Histological preparations were subjected to a visual analysis using an Olympus BX41 light microscope (Olympus 114 Corp., Tokyo, Japan) with Olympus DP12 digital camera (Olympus 114 Corp., Tokyo, Japan) and a PC computer and documented.

### Quantitative analysis

Fifteen (15) heart sections (three sections for H&E staining and three sections for each immunostaining, i.e. β-catenin, CacyBP/SIP, galectin-3 and LMP7 immunostaining) were examined from each man. Five randomly selected microscopic fields (each field 0.785 mm^2^, 200x magnification (20x lens and 10x eyepiece)) from each segment of the heart. Each obtained digital image of the heart cross-section was subjected to morphometric evaluation using NIS Elements AR 3.10 Nikon software for microscopic image analysis. In each analysed image, all cardiomyocytes were counted (only those cardiomyocytes with visible nucleus were considered); then the number of cardiomyocyte cells was converted and presented as mean values per 1 mm^2^ section area.

In each analysed image of the heart, the width of 25 randomly selected cardiomyocytes was measured and presented as mean values.

The intensity of the immunohistochemical reaction in each of the analyzed images was measured in 25 randomly selected areas of the positive signal. The intensity of immunohistochemical reaction was measured using a 0 to 255 grey scale level where the completely white or bright pixels were scored 0 and completely black pixels were scored 255.

### Real-time PCR

A heart sample (1 cm^3^), previously fixed in 10% buffered formalin solution and routinely embedded in paraffin, was taken from each patient. Total RNA was isolated using the High Pure FFPET RNA Isolation Kit (Roche) for formalin-fixed and paraffin-embedded tissues. Quantitative evaluation and quality control of total RNA was determined using a spectrophotometer—NanoDrop 2000 (ThermoScientific, Waltham, MA, USA). Only RNA samples for which the absorbance ratio at wavelength 260nm/280nm was 1.8–2.1 were adopted for the next analysis steps. The mentioned absorbance ratio proves that isolated RNA is of high quality An aliquot of 1μg of total RNA was reverse transcribed into cDNA using iScript^™^ Advanced cDNA Synthesis Kit for RT-qPCR (BIO-RAD, Berkeley, California, USA). cDNA synthesis was performed in a final volume of 20 μl using an Thermal Cycler (Model SureCycler 8800, Aligent Technologies). For reverse transcription, the mixtures were incubated at 46°C for 20 min then heated to 95°C for 1 min and finally rapidly cooled at 4°C.

Quantitative real-time PCR reactions were performed using) Stratagene Mx3005P (Aligent Technologies) with the SsoAdvanced^™^ Universal SYBER^®^ Green Supermix (BIO-RAD, Berkeley, California, USA). Specific primers for the β-catenin (*CTNNB1*), CacyBP/SIP (*CACY BP*), galectin-3 (*LGALS3*), LMP7 (*PSMB8*) and *GAPDH* were designed by BIO-RAD Company. The housekeeping gene *GAPDH* was used as the reference gene for quantification. In order to determine the amounts of tested genes expression levels, standard curves for each gene separately were constructed with serially diluted PCR products. PCR products were obtained by amplification of cDNA using specific primers as follows *CTNNB1* (qHsaCED0046518, BIO-RAD), *CACY BP* (qHsaCED0043669, BIO-RAD), *LGALS3* (qHsaCED0044416, BIO-RAD), *PSMB8* (qHsaCED0037294, BIO-RAD) and *GAPDH* (qHsaCED0038674, BIO-RAD). qRT-PCR was carried out in a dublete i.e. for each patient the expression of a gene in question was measured in two technical replicates. The qRT-PCR reaction was conducted in a final volume of 20 μl under the following conditions: 2 min polymerase activation at 95°C, 5 sec denaturation at 95°C, 30 sec annealing at 60°C for 35 cycles. PCR reactions were checked by including no-RT-controls, by omission of templates, and by melting curve to ensure only a single product was amplified. The relative quantification of gene expression was determined by comparison of values of Ct using the ΔΔCt method. All results were normalized to *GAPDH*.

### Statistical analysis

All data were analysed for statistical significance using software computer package Statistica Version 12.0. The mean values were computed automatically; significant differences were determined by Student’s t-test; p<0.05 was accepted as significant. The relationships between age and intensity of immunostaining for β-catenin, CacyBP/SIP, galectin-3, LMP7 as well as correlation between age and expression of β-catenin, CacyBP/SIP, galectin-3, LMP7 genes were examined using the Pearson's correlation coefficient, p<0.05 was considered statistically significant.

## Results

The results of the positive test with the antibodies used are shown in Fig 1.

Mean values of age, body weight and body mass index (BMI) in the two studied groups of men are presented in Table 1. There were no differences in mean body weight and BMI between subjects age over 45 years and younger participants (Table 1).

**Table 1:** Average age [years], body weight [kg] and BMI [kg/m^2^] in studied groups of men (mean ± SE).

Group of men	N	age [years]	weight [kg]	BMI [kg/m^2^]
men under 45 years old	6	37.5 ± 3.51	85.2 ± 2.58	26.1 ± 0.54
Min		23	80	24.2
Max		45	95	27.7
men over 45 years old	6	56.5 ± 2.89 *	85.2 ± 2.12	26.4 ± 0.54
Min		46	80	24.8
Max		65	95	28.7

* p < 0.05 men over 45 vs men under 45

**Fig 2:**
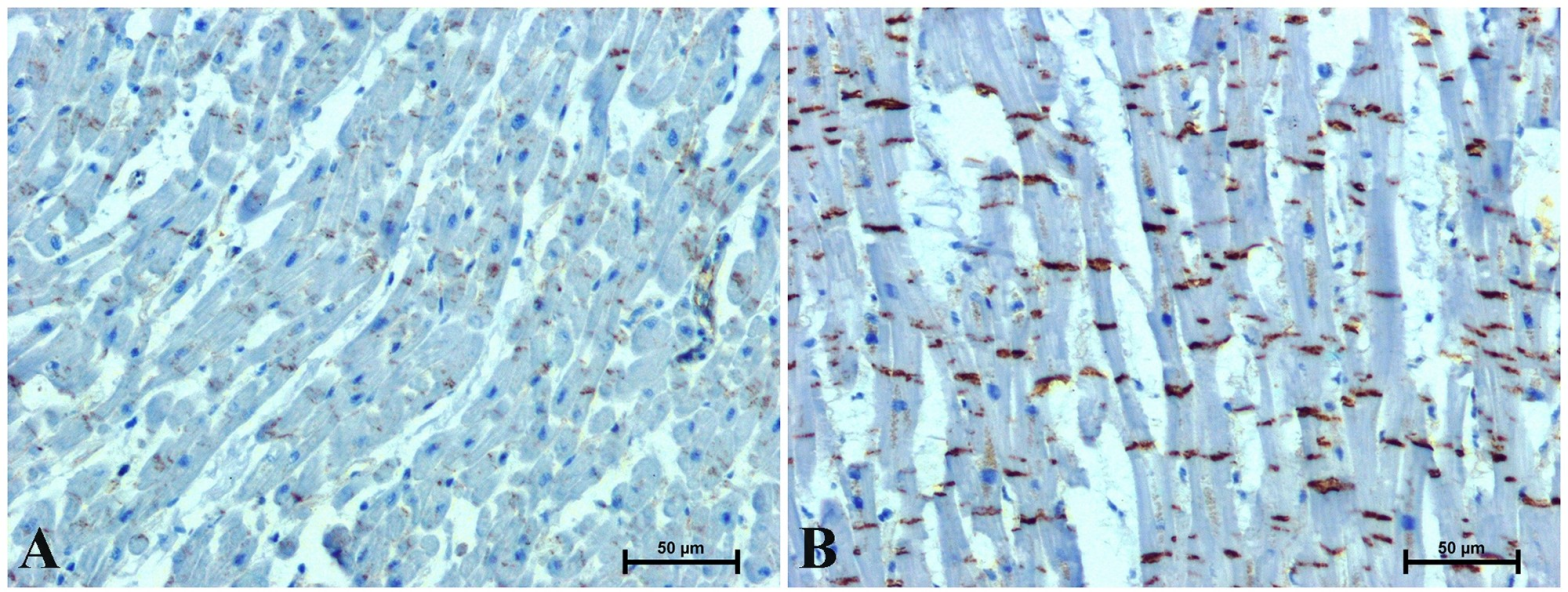
Representative images of immunoreaction determining β-catenin in the heart of men under 45 (A) and over 45 (B) years old. (Number of analysed images for each subject = 15, Total number of analysed images for each group of men N = 90).

**Fig 3:**
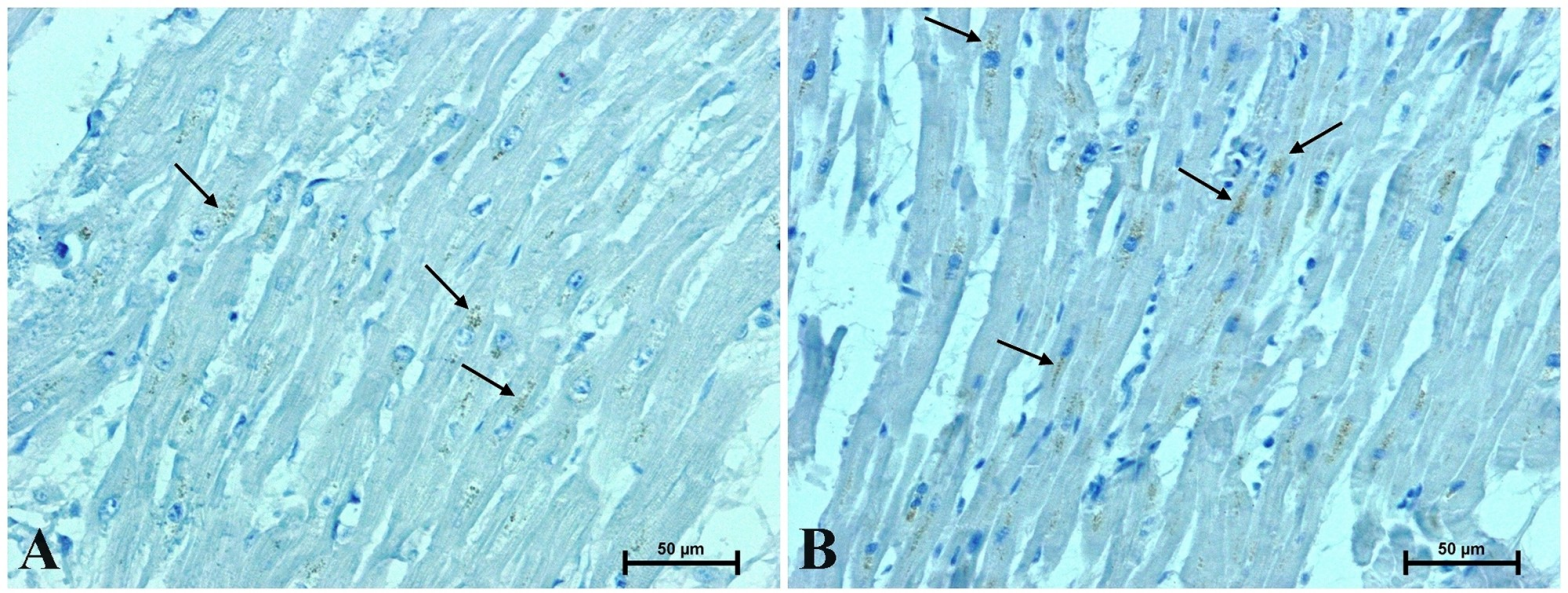
Representative images of CacyBP/SIP-immunostaining in the heart of men (arrows): (A) under 45 and (B) over 45 years old. (Number of analysed images for each subject = 15, Total number of analysed images for each group of men N = 90).

**Fig 4:**
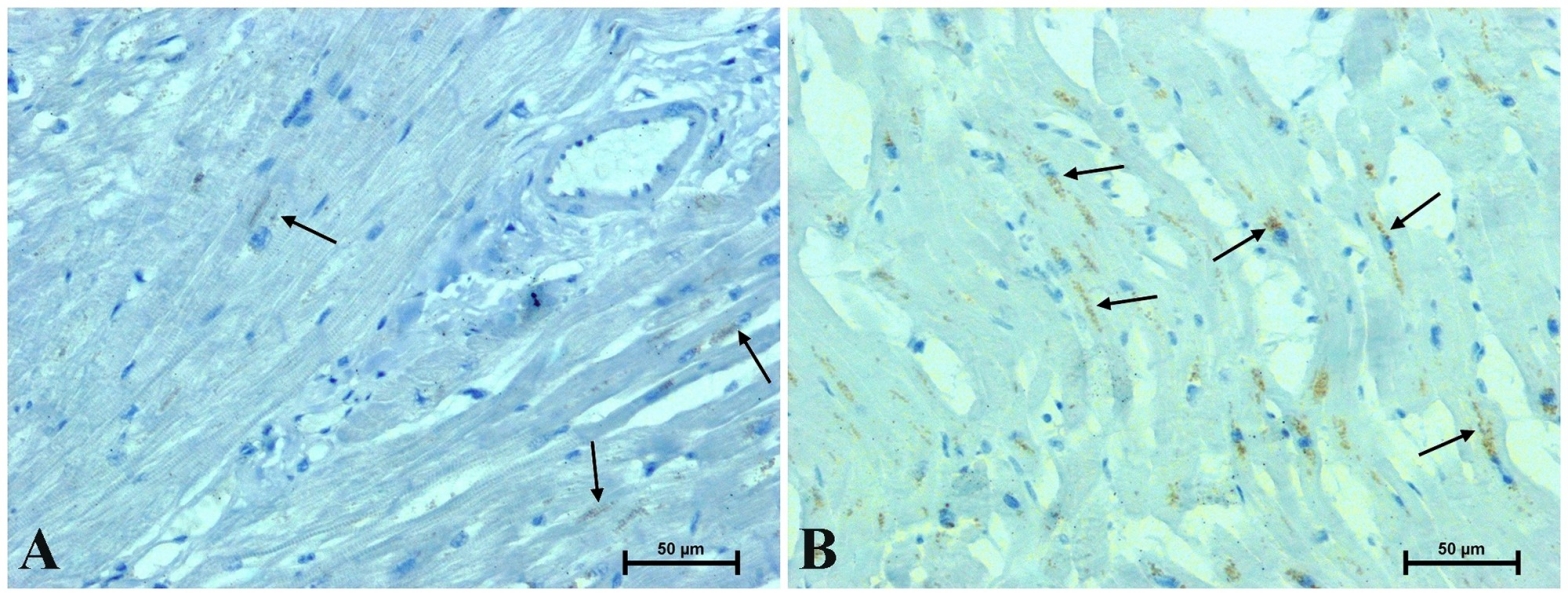
Representative images of immunohistochemical identification of galectin-3 in the heart of men (arrows): (A) under 45 and (B) over 45 years old. (Number of analysed images for each subject = 15, Total number of analysed images for each group of men N = 90).

**Fig 5:**
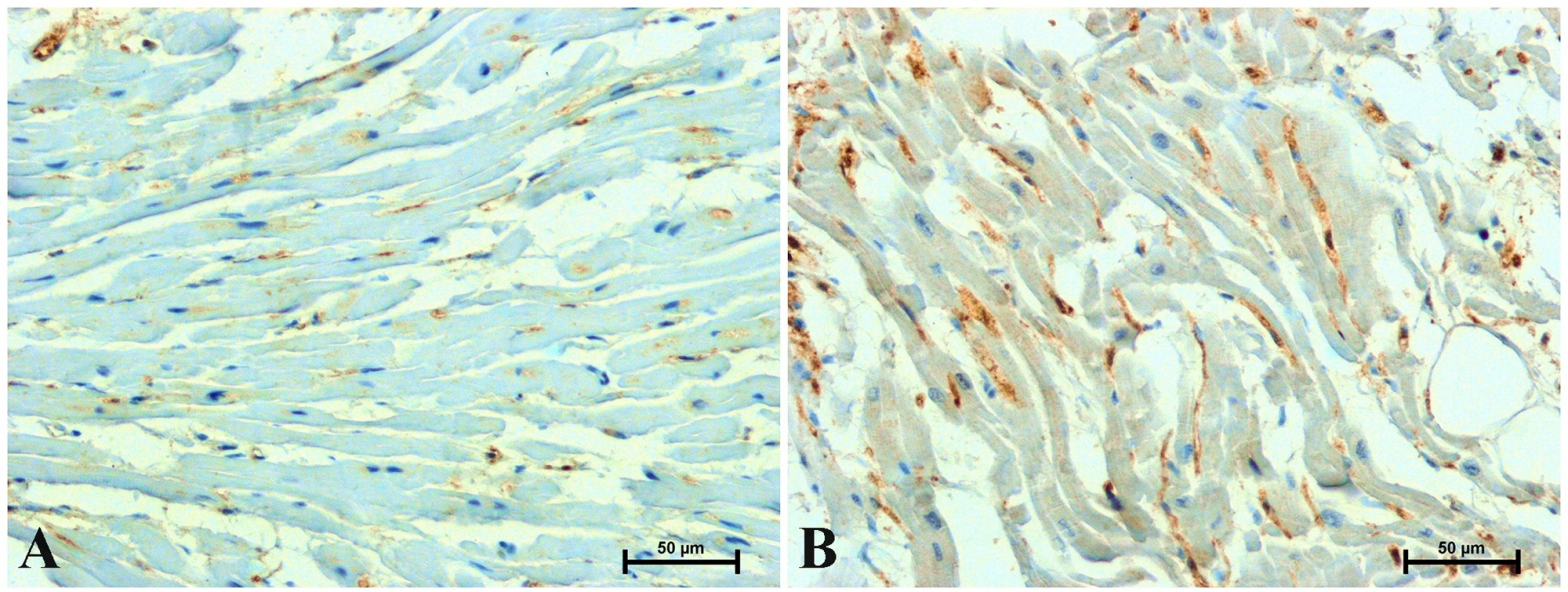
Representative images of LMP7 immunodetection in the heart of men: (A) under 45 and (B) over 45 years old. (Number of analysed images for each subject = 15, Total number of analysed images for each group of men N = 90).

During a routine histological assessment, no significant pathological changes were found in the hearts of the studied men. Positive immunohistochemistry with β-catenin, CacyBP/SIP, galectin-3 and LMP7 was observed in the hearts of all the men tested, although reaction intensity varied between different age groups (Figs 2–5 and Table 2).

**Table 2:** Intensity of immunoreaction against β-catenin, CacyBP/SIP, galectin-3 and LMP7 immunoproteasome subunit in men heart (mean ± SE). The correlation between age and intensity of immunohistochemical staining for β-catenin, CacyBP/SIP, galectin-3, LMP7.

Group of patients	N of measurements	Intensity of immunoreaction in men heart Scale from 0 (white pixel) to 255 (black pixel)					
		β-catenin		CacyBP/SIP	galectin-3	LMP7	
		intercalated discs	cardiomyocytes cytoplasm			cardiomyocyte cytoplasm	interstitial cells
**men under 45 years**	2250	112.4 ± 1.93	66.4 ± 1.71	71.2 ± 3.36	78.6 ± 1.81	73.5 ± 1.62	170.3 ± 2.43
Min		79.1	36.4	40.4	55.1	52.6	148.9
Max		138.0	98.2	122.3	100.6	95.2	200.1
**men over 45 years**	2250	180.8 ± 2.33*	96.4 ± 1.24*	107.3 ± 3.47*	89.3 ± 2.43*	109.1 ± 2.40*	158.5 ± 1.90*
Min		147.6	78.9	68.1	63.1	73.3	158.5
Max		211.0	114.2	157.8	121.5	143.2	211.3
**Correlation coefficients between age and intensity of immunohistochemical staining**		0.947^♯^	0.933^♯^	0.864^♯^	0.737^♯^	0.899^♯^	0.783^♯^

* p < 0.05 men over 45 vs men under 45 years old

^♯^ correlation is significant at the 0.05 level

Immunostaining of β-catenin in the hearts of men revealed its location in intercalated discs and in the perinuclear area of cardiomyocytes cytoplasm (Fig 2A and 2B). In the hearts of men under 45 years of age was noted moderate, rarely strong intensity of β-catenin immunostaining in the intercalated discs and delicate near cardiomyocyte nuclei (Fig 2A). Much stronger immunoreactivity for β-catenin, both in the perinuclear region of cardioniocyte cytoplasm and in intercalated discs was found in the hearts of patients over 45 years of age (Fig 2B).

CacyBP/SIP was identified in the man’s hearts in the form of small brown-stained granules located near the cardiomyocyte nuclei (Fig 3A and 3B). CacyBP/SIP immunoreactivity in the hearts of men under 45 years of age was weak (Fig 3A), whereas in the myocardium of older men a much higher intensity of the CacyBP/SIP reaction was noted (Fig 3B).

Antibodies against galectin-3 gave a very weak or almost undetectable reaction in the myocardium of men under 45 (Fig 4A), whereas in the hearts of older men, a fairly intense galectin-3 immunoreaction in the perinuclear area of cardiomyocytes was observed (Fig 4B).

**Fig 6:**
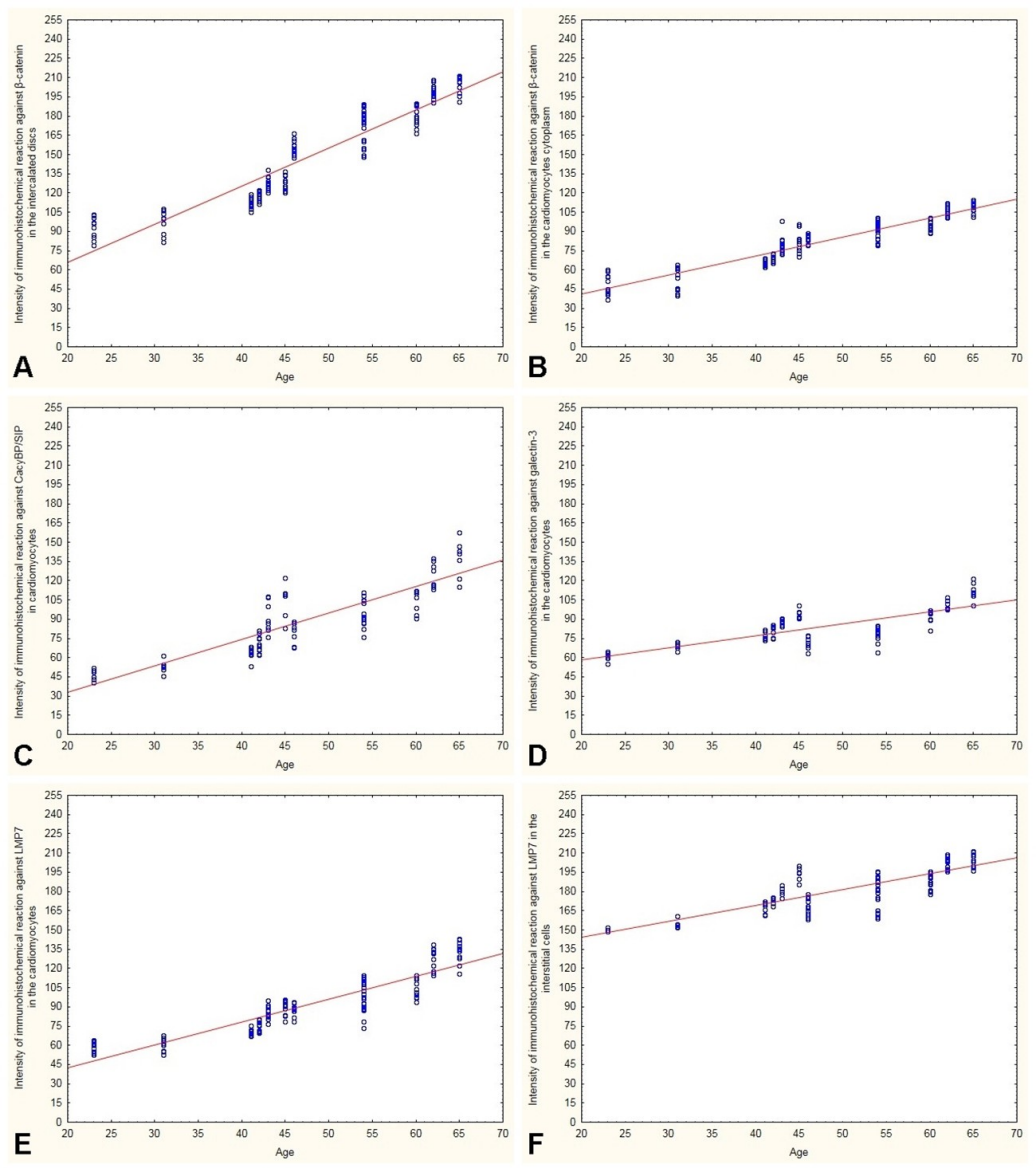
Relationship between age and intensity of immunohistochemical reaction in the man’s heart: Immunoreaction against β-catenin in intercalated discs (A), immunoreaction against β-catenin in cardiomyocytes cytoplasm (B), immunoreaction against CacyBP/SIP in cardiomyocytes (C), immunoreaction against galectin-3 in cardiomyocytes (D) immunoreaction against LMP7 in cardiomyocytes (E), immunoreaction against LMP7 in the interstitial cells (F).

Immunodetection of LMP7 in the hearts of men under 45 gave a delicate to moderate immunostaining in the cytoplasm of myocardial cells and a strong reaction in several interstitial cells (Fig 5A). The intensity of the reaction was far stronger in the hearts of men over 45 years old. Clusters of cardiomyocytes with intense cytoplasmic immunoreactivity of LMP7 and more interstitial cells with strong LMP7 immunostaining were observed in these hearts (Fig 5B). The results of densimetric tests confirmed significantly increased intensity of immunohistochemical reactions for β-catenin, CacyBP/SIP, galectin-3 and LMP7 in the hearts of men over 45 years of age compared to younger individuals (Table 2). A positive correlation was found between age and the intensity of immunohistochemical reactions for β-catenin, CacyBP / SIP, galectin-3 and LMP7 in the heart of the examined men (Table 2, Fig 6.). Computer image analysis demonstrated a smaller number of myocardial cells (60.5 ± 0.56 cardiomyocytes per 1 mm^2^ section area) and increased width of cardiomyocytes (cardiomyocytes width 11.1 ± 0.28 μm) in the hearts of men over 45 years of age compared with younger men (69.7 ± 1.12 cardiomyocytes per 1 mm^2^ section area, cardiomyocytes width 9.2 ± 0.29 μm) (Table 3).

**Table 3:** The number of cardiac muscle cells per 1 mm^2^ section area and width of cardiomyocytes in men heart (mean ± SE).

Group of men	N of measurements	The number of cardiac muscle cells per 1 mm^2^ section area	N of measurements	Width of cardiomyocytes [μm]
**men under 45 years old**	90	69.7 ± 1.12	2250	9.2 ± 0.29
min		60		7.5
max		81		12.4
**men over 45 years old**	90	60.5 ± 0.56 *	2250	11.1 ± 0.28 *
min		57		8.5
max		66		14.5

* p < 0.05 men over 45 vs men under 45 years old

**Fig 7:**
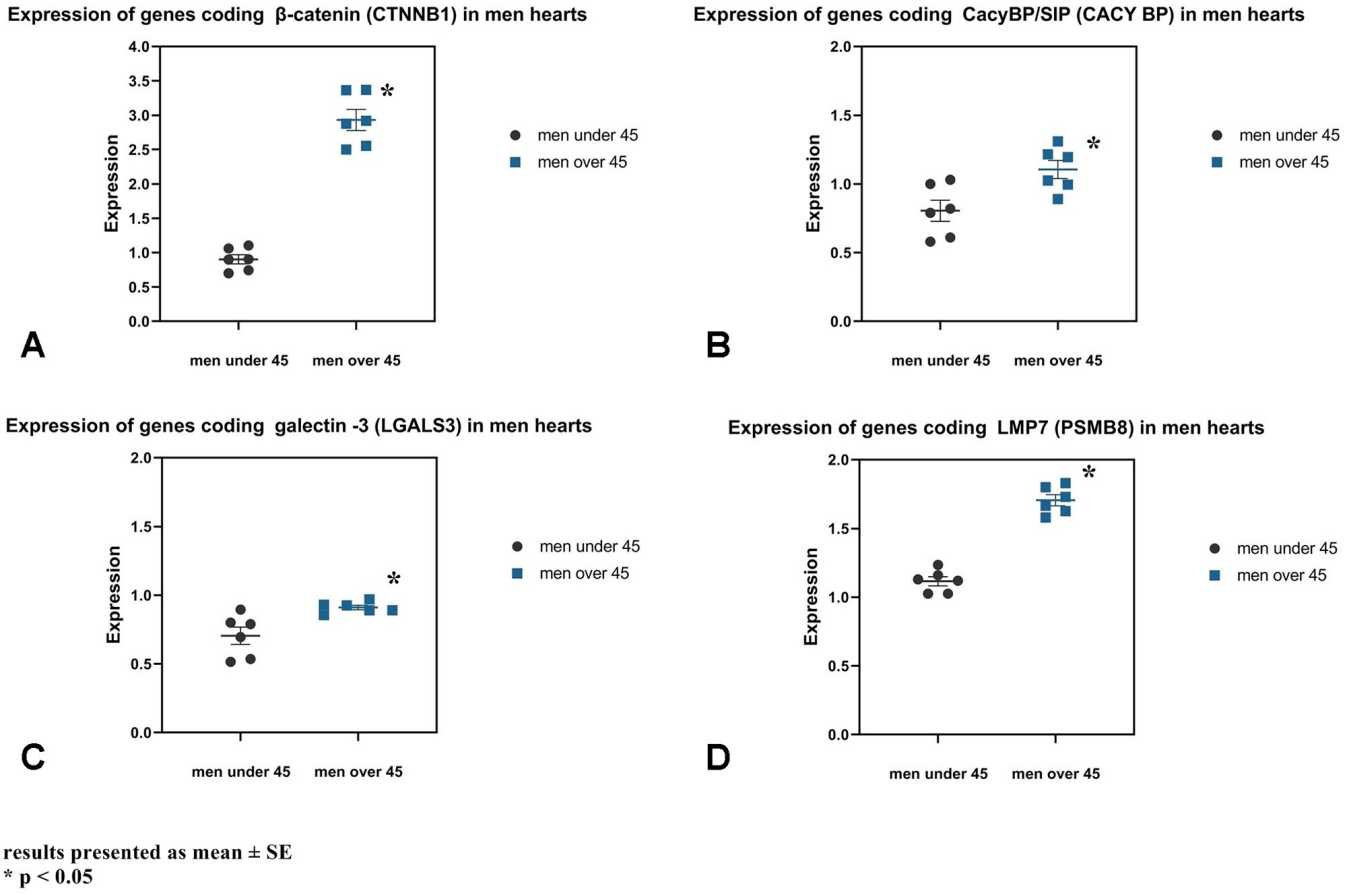
Comparison of expression of genes coding for β-catenin (CTNNB1) (A), CacyBP/SIP (B), galectin-3 (LGALS3) (C) and LMP7 (PSMB8) (D) in the heart of men at different age.

**Fig 8:**
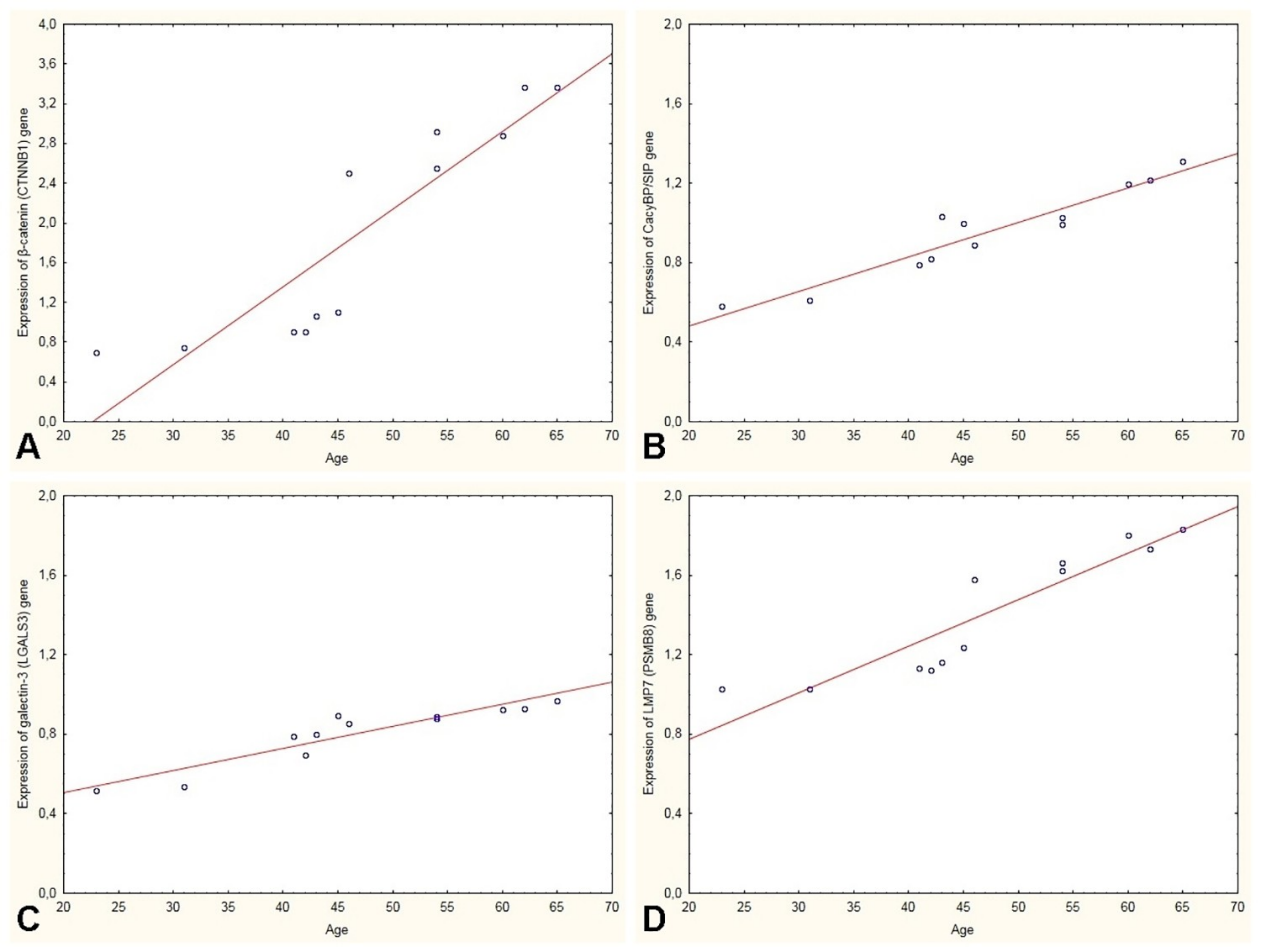
Relationship between age and expression of genes coding β-catenin (CTNNB1) (A), CacyBP/SIP (B), galectin-3 (LGALS3) (C) and LMP7 (PSMB8) (D) in the men heart.

A test utilising qRT-PCR method showed an increase in the expression of β-catenin, CacyBP/SIP, galectin-3 and LMP7 genes in the heart of men over 45 years of age compared to men under 45 years of age (Fig 7, Table 4). The expression of the β-catenin, CacyBP/SIP, galectin-3 and LMP7 genes in the man hearts was positively correlated with the age individual’s (Table 4, Fig 8).

**Table 4:** Expression of genes coding for β-catenin, CacyBP/SIP, galectin-3 and LMP7 in men hearts (mean ± SE). The correlation between expression of gene coding for β-catenin, CacyBP/SIP, galectin-3, LMP7 and individual’s age.

Group of patients	N (measured in two technical replicates)	Expression of genes			
		β-catenin (CTNNB1)	CacyBP/SIP	galectin-3 (LGALS3)	LMP7 (PSMB8)
**men under 45 years old**	**6**	0.9 ± 0.05	0.8 ± 0.21	0.7 ± 0.18	1.1 ± 0.09
**Min**		0.67	0.55	0.49	0.98
**Max**		1.13	1.05	0.91	1.25
**men over 45 years old**	**6**	2.9 ± 0.10 *	1.1 ± 0.19 *	0.9 ± 0.02 *	1.7 ± 0.11 *
**Min**		2.48	0.87	0.85	1.55
**Max**		3.42	1.33	0.99	1.85
**Correlation coefficients between gene expression and individual’s age**		0.896 ^♯^	0.931 ^♯^	0.896 ^♯^	0.913 ^♯^

* p < 0.05 men over 45 vs men under 45 years old

^♯^ correlation is significant at the 0.05 level

## Discussion

Ageing is one of the most significant risk factors for development of cardiovascular complications and mortality due to cardiovascular events [1, 2]. After the age of 45, the risk of cardiovascular diseases and cardiovascular mortality increases progressively with each decade of life [38]. Enhancing our knowledge of the mechanisms that contribute to structural and functional changes related to age in the heart is necessary to increase the effectiveness of prevention and treatment of cardiomyopathy in the elderly. This prompted us to undertake the research, aimed at comparative evaluation of the distribution and expression of β-catenin, CacyBP/SIP, galectin-3 and LMP7 in the hearts of healthy men under and above 45 years of age.

As evident from the review of the literature, this is the first report on the comparative evaluation of the distribution of β-catenin, CacyBP/SIP, galectin-3 and LMP7 immunosubunit in the hearts of healthy men in different age groups. In the present study, we found a stronger immune response against β-catenin, CacyBP/SIP, galectin-3 and LMP7 in the hearts of men over 45 years of age compared to younger individuals.

The present report also revealed increased, age-related, expression of the β-catenin, CacyBP/SIP, galectin-3 and LMP7 gene in the hearts of men over 45 years of age, which was consistent with immunohistochemical changes in peptide content.

Our findings are consistent with experimental data showing increased levels of β-catenin in the heart and enhanced production of immunoproteasomes in skeletal muscles of ageing rodents [31, 35]. Several previous clinical trials have also demonstrated a positive correlation between galectin-3 plasma concentration and human age [24, 25]. Due to the difficulty with obtaining human material, there are no data illustrating changes in the content of β-catenin and galectin-3 or proteasomal activity in the myocardium of older individuals.

Ageing is accompanied by histopathological changes in the heart, such as a successive loss of cardiomyocytes, myocardial hypertrophy and fibrotic remodelling of the heart [1, 39].

β-catenin, CacyBP/SIP and galectin-3 are crucial for the survival of cardiomyocytes. *In vitro* studies by Hahn et al, [3], Au et al. [17] and Bergmann et al. [40] have revealed that β-catenin and CacyBP/SIP have a protective effect on cardiomyocytes in conditions of serum deficiency or hypoxia/reoxygenation. Analogously, studies on rats have indicated that β-catenin reduces the apoptosis of cardiac muscle cells in the model of myocardial infarction [3]. In contrast, Lin et al, [5] demonstrated that genetic enhancement expression of β-catenin in cultured cardiomyocytes results in suppression of survival pathway markers and induced cell apoptosis. Galectin-3 has been shown to negatively affect myocardial cell viability, increasing proapoptotic factors and promoting oxidative stress [27]. Exposure of cultured cardiomyocytes to cadmium and doxazosin resulted in elevated levels of cellular galectin-3 [41, 42]. Considering the above and observed herein increase in content and expression of genes encoding β-catenin, CacyBP/SIP and galectin-3 in the hearts of men over 45 years, it might be suspected that those proteins are possibly implicated in process of age-related death of cardiomyocytes.

Some recent evidence underlines the profibrotic and hypertrophic effects of β-catenin and galectin-3 in the heart and shows on deleterious consequence of activating those peptides on cardiomyocytes. The modification of β-catenin gene, resulting in the expression of the non-degradable peptide form and its accumulation in the cytoplasm, provoked enlargement of isolated rat cardiomyocytes [10]. Similarly, genetic stabilization of β-catenin in mice caused cardiac hypertrophy in those animals [10]. Jiang et al. [12] showed that pharmacological inhibition of WNT/β-catenin signalling attenuated cardiac hypertrophy and fibrosis, reduced cardiac dysfunction, and animals mortality in a mouse model of pressure overload heart hypertrophy. Hirschy et al. [43] demonstrated that inactivation of β-catenin gene in mice did not affects the animal phenotype and was well tolerated, however, stabilization of β-catenin lead to a dramatic increase in cardiomyocyte size, cardiac fibrosis, dilated cardiomyopathy and, mice death within 5 months of age. Zelarayán et al. [11] showed that genetic depletion of β-catenin weakened fibrotic and hypertrophic changes in the heart, significantly improved left ventricular function, and enhanced survival of mice undergoing myocardial infarction, while genetic stabilization of β-catenin had opposite effects. The intrapericardial infusion of galectin-3 induced cardiac fibrosis, cardiomyocyte hypertrophy and impairment of heart function in rats [44, 45], whereas genetic or pharmacological inhibition of galectin-3 limited the deposition of collagen and prevented cardiac dysfunction in several animal models of heart disease [46]. In light of the above, the results of our study might indicate a potential relationship between increased levels of β-catenin and galectin-3 in the heart of older men and age-related remodelling of the heart, as well as the possible implication of β-catenin and galectin-3 in progressive cardiac dysfunction with age.

In our study, we observed significantly higher content of β-catenin in the intercalated discs in the heart of older men compared to those under the age 45 years. The presented results are consistent with a report by Bonda et al. [35] who found an increased level of β-catenin in the myocardium of older mice. β-catenin coordinates the formation of gap and adherens junctions in the intercalated discs and therefore it is crucial for maintaining the mechanical integrity of heart tissue [6, 8, 35]. It regulates intercellular connections through the interaction with connexin 43 and N-cadherin [6, 8, 35]. Ageing proceeds with significant disorganisation of the structure of the intercalated discs. This applies to the expansion of the intercellular space between cardiomyocytes, disturbances in the structure of adherens junctions, reduction of the density of gap junctions [35]. Our research demonstrated the enhanced expression of the β-catenin gene and accumulation of this peptide in the intercalated disc in the heart of men over 45 years of age, which given the role of β-catenin in coordination of structure of the intercalated discs, might suggest the possible involvement of β-catenin in maintaining of mechanical and electrical communication between cardiomyocytes in the ageing heart.

Oxidative stress is considered to be one of the major pathomechanisms associated with the process of cell ageing and apoptosis [28]. Topolska-Woś et al. [47] and Góral et al. [48] have demonstrated that the induction of oxidative stress results in increased CacyBP/SIP expression in various cell lines. Perhaps the presented in our report the increase in gene expression and CacyBP/SIP level in the hearts of men over 45 years might be associated with disturbance of oxidative balance in aging cardiomyocytes.

The latest literature data indicate a significant impairment of proteasomes activity in the ageing heart [28, 29]. It has been hypothesized that reduced proteolytic capacity of proteasomes in aging cardiomyocytes may be associated with their oxidative modification [28, 29]. At the same time, aging is associated with alterations in the body’s immune system and a significant increase in the level of circulating cytokines, such as tumour necrosis factor α (TNF-α) and interleukin 6 (IL-6) [49]. Those cytokines induce the production of specific inducible subunits and the conversion of constitutive proteasomes into immunoproteasomes [31, 32]. Husom et al. [31] demonstrated significant modifications in proteasome composition and a substantial increase in LMP7 immunoproteasomes in the skeletal muscles of older rats. Increased gene expression and LMP7-immmunoreactivity in the hearts of older men found in our study may be associated with a similar transformation of constitutive proteasomes to immunoproteasomes.

The results of quantitative and morphometric analyses revealed a decrease in the number of cardiomyocytes and the hypertrophy of cardiac muscle cells in men older than 45 years. In a study on the impact of aging on the human heart, Olivetti et al. [39] also observed a gradual decrease in the number of myocardial cells and hypertrophy of the remaining cardiomyocytes in the hearts of older men. It can be assumed that due to a loss of cardiomyocytes caused by aging, the remaining cells are subjected to greater stress and consequently are subject to compensatory hypertrophy in response to an increased workload.

Our research results clearly show that the aging process leads to disruption in the expression and content of β-catenin, CacyBP/SIP, galectin-3 and immunoproteasomes in heart cells.

To the best of the authors' knowledge, this is the first study characterizing the hearts of healthy men for their expression of β-catenin, CacyBP/SIP, galectin-3 and immunoproteasome subunit LMP7. The differences in the expression of the examined parameters in the heart of men of different ages, demonstrated in the conducted studies, suggest participation of β-catenin, CacyBP/SIP, galectin-3 and LMP7 in the regulation of many important processes and heart homeostasis during ageing.

## Supporting information

S1 Fig (TIF)
