# Supplementary figures and images for: Ageing-related changes in the levels of β-catenin, CacyBP/SIP, galectin-3 and immunoproteasome subunit LMP7 in the heart of men
Source: PLoS One. 2020 Mar 2;15(3):e0229462. doi: 10.1371/journal.pone.0229462 (PMC7051089; doi:10.1371/journal.pone.0229462)

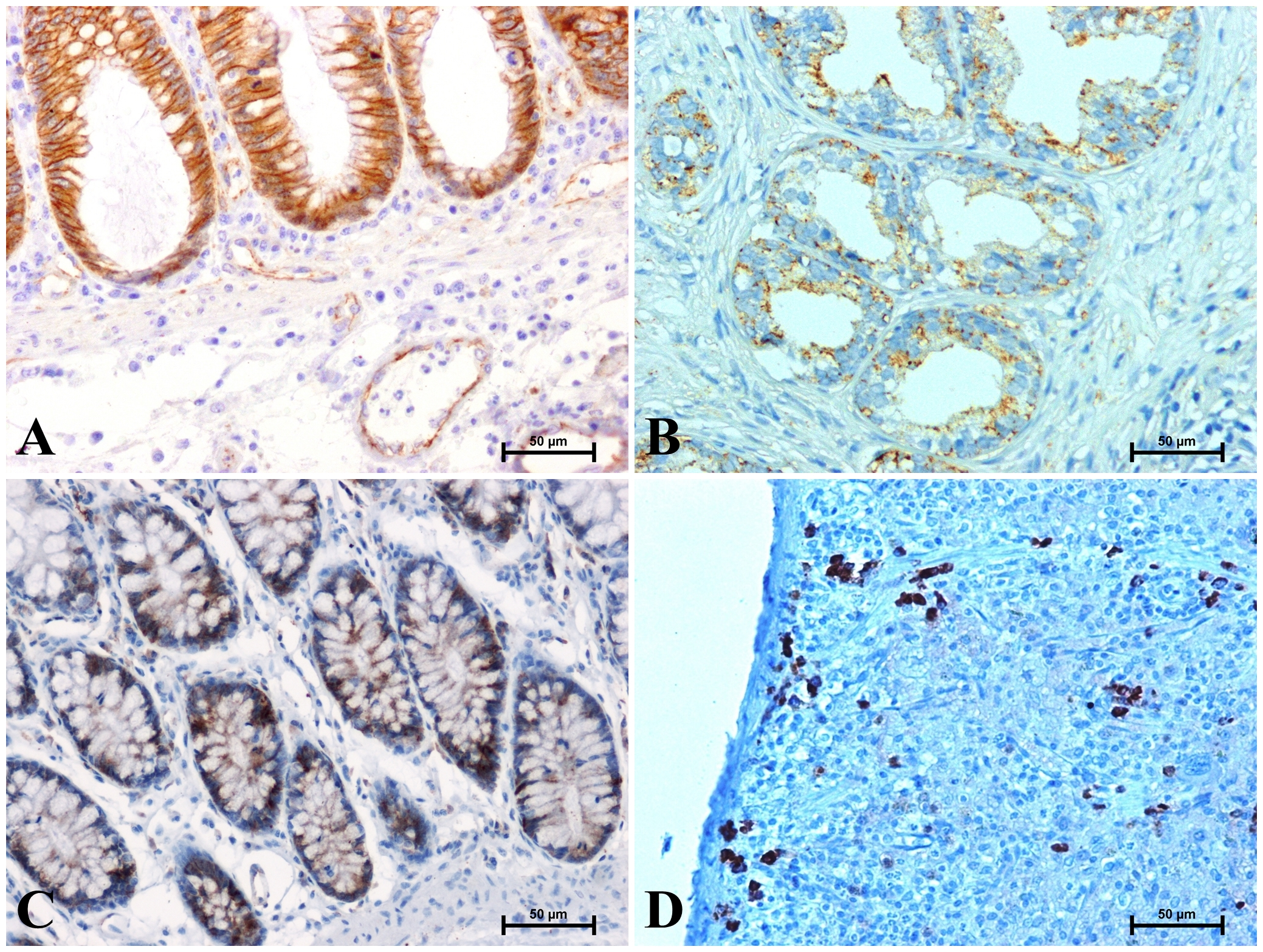

Supplement: S1 Fig — (TIF) [file pone.0229462.s001.tif]
